# Insulin receptor signaling and glucagon-like peptide 1 effects on pancreatic beta cells

**DOI:** 10.1371/journal.pone.0181190

**Published:** 2017-08-02

**Authors:** Nunzia Caporarello, Cristina Parrino, Vincenzo Trischitta, Lucia Frittitta

**Affiliations:** 1 Endocrine Unit, Department of Clinical and Experimental Medicine, University of Catania, Catania, Italy; 2 IRCCS Casa Sollievo della Sofferenza, Research Unit of Diabetes and Endocrine Diseases, San Giovanni Rotondo, Italy; 3 Department of Experimental Medicine “Sapienza” University, Rome, Italy; Consiglio Nazionale delle Ricerche, ITALY

## Abstract

Glucagon-like peptide-1 (GLP-1) is a potent gluco-incretin hormone, which plays a central role on pancreatic beta cell proliferation, survival and insulin secreting activity and whose analogs are used for treating hyperglycemia in type 2 diabetes mellitus. Notably, abnormal insulin signaling affects all the above-mentioned aspects on pancreatic beta cells. The aim of our study was to investigate whether the protective effects of GLP1-1 on beta cells are affected by altered insulin receptor signaling. To this end, several effects of GLP-1 were studied in INS-1E rat beta cells transfected either with an inhibitor of insulin receptor function (i.e., the Ectonucleotide Pyrophosphatase Phosphodiesterase 1, ENPP1), or with insulin receptor small interfering RNA, as well as in control cells. Crucial experiments were carried out also in a second cell line, namely the βTC-1 mouse beta cells. Our data indicate that in insulin secreting beta cells in which either ENPP1 was up-regulated or insulin receptor was down-regulated, GLP-1 positive effects on several pancreatic beta cell activities, including glucose-induced insulin secretion, cell proliferation and cell survival, were strongly reduced. Further studies are needed to understand whether such a scenario occurs also in humans and, if so, if it plays a role of clinical relevance in diabetic patients with poor responsiveness to GLP-1 related treatments.

## Introduction

Glucagon-like peptide 1 (GLP-1) is a potent gluco-incretin hormone secreted from the enteroendocrine L cells in response to food ingestion [1], which exerts a positive effect on insulin secretion, beta cell proliferation and apoptosis [2, 3]. Based upon this main physiological role, incretin-based therapies have become an attractive tool for treating hyperglycemia in patients with type 2 diabetes mellitus. Unfortunately, up to 60% patients are unresponsive to such therapies for so far unknown reasons [4–8]. Several studies in animal models have consistently reported that abnormal insulin signaling affects insulin secretion, proliferation and survival of beta cells [9–11]. Along the same line of evidences, human non-synonymous genetic polymorphisms (i.e. ENPP1 K121Q, IRS-1G972R and TRIB3Q84R) affecting insulin signaling pathway [12–15] are able to reduce, both as singly considered and even more in combination, insulin secretion *in vivo* [16], in isolated human islets [16–19] and in cultured beta-cells [18–21]. Thus, an intriguing scenario has emerged suggesting that abnormalities impairing insulin signaling play a role on glucose homeostasis not only by reducing insulin sensitivity in peripheral tissues (i.e. liver and skeletal muscle), but also by affecting several aspects of beta cells functionality [20–21].

Several studies have shown that there is a cross talk between G-protein coupled receptors, including GLP-1 receptor, and tyrosine-kinase receptors, including insulin receptor [22–24].

Whether in beta cells the protective effect of GLP-1 on insulin secretion, proliferation and survival is affected by abnormal insulin signaling is a fascinating possibility with potential clinical relevance, which has never been addressed.

To answer this question, rat and mouse cultured beta cells were manipulated either by up-regulating ENPP1, a known inhibitor of insulin receptor signaling [21, 25] or by down-regulating insulin receptor itself.

## Materials and methods

### Antibodies and reagents

Glucagon-like peptide-1 (7–36) amide and antibody against actin were obtained from Sigma Aldrich (St. Louis, MO, USA).

Antibodies anti-phospho-44/42-mitogen-activated protein kinase (ERK 1/2), anti total ERK 1/2, anti phospho-AKT, anti total AKT, anti-ENPP1 and anti-Insulin Receptor were purchased from Cell Signaling Technology (New England Biolabs, Beverly, MA). Anti GLP1-R antibodies were obtained from Santa Cruz Biotechnology (Santa Cruz, CA, USA).

All other chemicals were of the highest grade commercially available.

### Cell culture

Rat insulin-secreting INS-1E cells (a kind gift from C. B. Wollheim, Department of Cell Physiology and Metabolism, University of Geneva, Geneva, Switzerland) were grown in monolayer cultures in regular RPMI 1640 medium supplemented with 10% heat-inactivated Fetal Bovine Serum (FBS), 10 mmol/l HEPES, 100 IU/ml penicillin, 100μg/ml streptomycin, 1 mmol/l sodium pyruvate, 2 mmol/l L-glutamine and 50μmol/l ß-mercaptoethanol in a humidified atmosphere (5% CO_2_/95% air) at 37°C. βTC-1 beta cell line, derived from transgenic mouse insulinoma, was grown in Dulbecco’s modified Eagle’s medium containing 25 mmol/l glucose supplemented with 15% horse serum (HS), 2.5% heat inactivated FBS, 1 mmol/l sodium pyruvate, 100 IU/ml penicillin, 100μg/ml streptomycin, 2 mmol/l L-glutamine and 50μmol/l ß-mercaptoethanol in a humidified atmosphere (5% CO_2_/95% air) at 37°C. In studies involving serum-starvation, serum was replaced by 0.1% BSA in medium containing 3 mmol/l glucose. We conducted all the experiments in starvation because both INS-1E and βTC-1 are insulinoma-derived cells, constitutively producing insulin. As a matter of fact, in these cells starvation reduced, but not abolished, insulin secretion, with insulin concentration in the medium being in the range of 0.8–1.0 nmol/L.

### Plasmid and transfections

**Fig 1:**
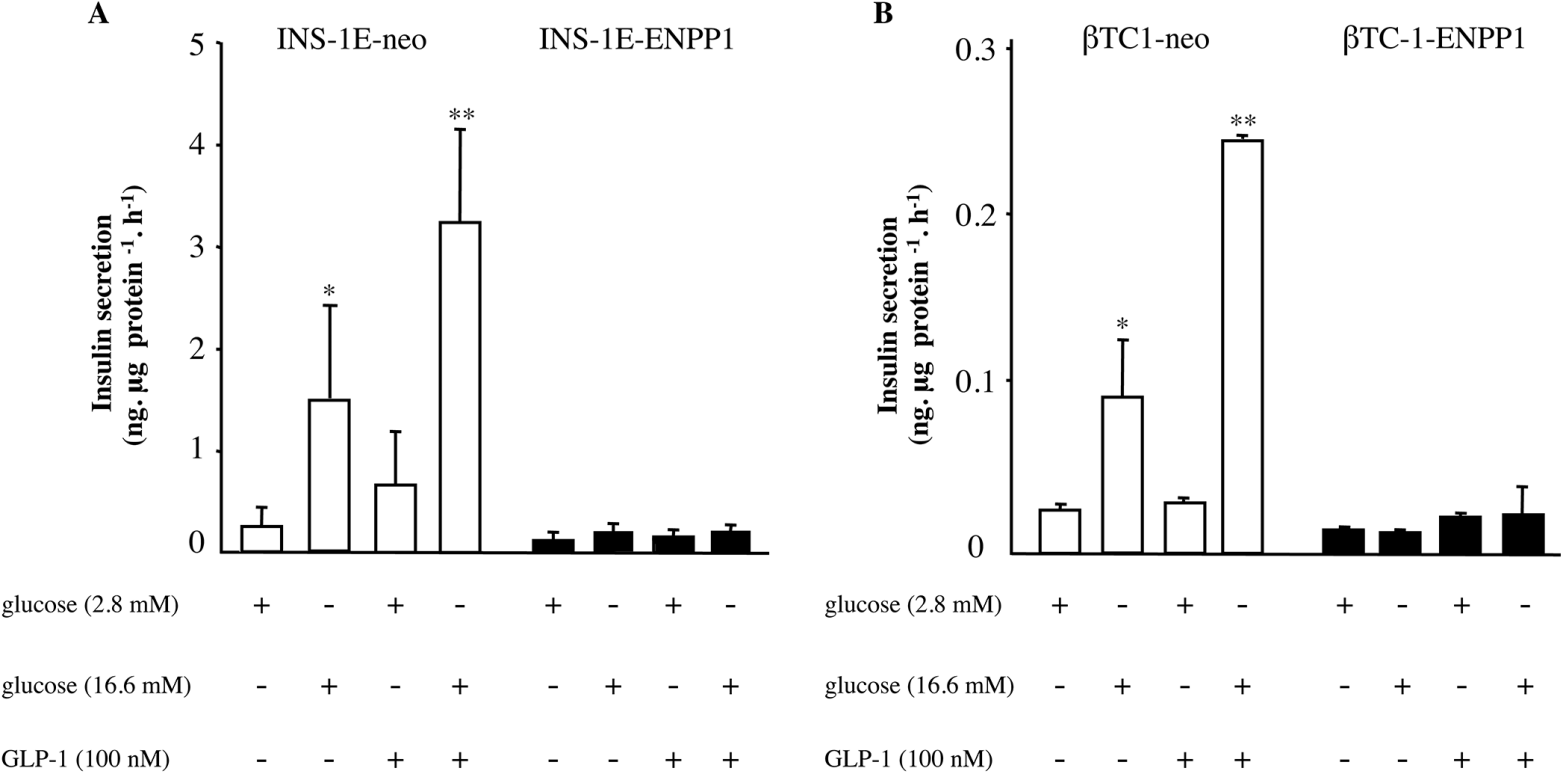
GLP-1 fails to increase insulin secretion in ENPP-1 transfected cells. INS-1E (panel A) and βTC-1 (panel B) cells, either transfected with neomicine (neo) or ENPP1 gene variant, were exposed to 100 nmol/l GLP-1 (60 min, 37°C) and glucose induced insulin secretion measured. Values are expressed as means ± S.D. of five independent experiments; *p<0.05 compared to neo-transfected cells at 2.8 mmol/l glucose; **p<0.05 compared to neo cells at 16.6 mmol/l glucose.

The full-length cDNA of ENPP1-Q121 was generated by site directed mutagenesis as previously described [13] and cloned in mammalian expression vector pRK7 [13]. INS-1E cells were either transfected with a plasmid (pRK7-neo) containing the neomycin resistance gene (INS1E-neo) or with the pRK7-neo plus the ENPP1-Q121 cDNA (INS-1E-ENPP1) by using the Fugene Transfection Reagent (Roche Germany) [19] and a clone overexpressing ENPP-1 (i.e. INS-1E-ENPP1) was selected (Fig 1A, S1 Fig). Transient transfections with pRK7-neo (βTC1-neo) and ENPP1-Q121 (βTC1-ENPP1) were carried out in βTC-1 cells (Fig 1B, S1 Fig).

The insulin receptor knock-down in INS-1E cells was obtained by small interfering RNA (siRNA) transfection. A pool of 3 different siRNAs was transfected in INS1-E cells for insulin receptor RNA interference, by using Lipofectamine RNAiMax (Life Technologies). A no targeting siRNA (scramble) was used as negative control, according to the manufacturer’s instruction. A significant down-regulation of insulin receptor expression (83±5% reduction) was obtained, as compared to scramble control cells (Fig 1C, S1 Fig).

### Glucose induced insulin secretion

Glucose induced insulin secretion was evaluated as previously described [19, 25] Briefly, 3x10^5^ cells/well were seeded in 24-well plates and grown for 72 hours. The day of the experiment, cells were washed twice with glucose-free Krebs solution pH 7.4 and pre-incubated (1 hour, 37°C, 5% CO_2_) in Krebs buffer containing 2.8mmol/l glucose. Insulin secretion was determined in the presence of 2.8 mmol/l or 16.6 mmol/l glucose in the absence or presence of GLP-1 (100 nmol/l). After 60 minutes at 37°C, aliquots of supernatant were taken for measuring insulin concentration, while total protein content was determined by using BCA protein assay (Pierce, Rockford, IL, USA), as previously described [19]. Rat/mouse insulin was measured by ELISA (Millipore, Billerica, MA, U.S.A).

### Cell proliferation

Briefly, a triplicate of 10^4^ cells/well was seeded in 24-well plates and grown for 72 hours. Cells were switched to serum free medium for 12 hours and then incubated for 48 hours in the absence or presence of GLP-1 (100 nmol/l). Cell proliferation was assessed by measuring DNA synthesis in proliferating cells by the pyrimidine analog 5-bromo-2’-deoxyuridine (BrdU) incorporation assay (Perkin Elmer, Waltham, MA, USA). BrdU labeling was performed for the 48-hour incubation period in the presence or absence of GLP-1 (100 nmol/l).

### Apoptosis

Apoptosis was measured by the caspase-3/7 activity. Cells (10^*4*^ cells/well) seeded in 96-well plates in triplicate and grown for 72 hours, were switched to serum free medium for 12 hours and then incubated (2 hours at 37°C) with staurosporine (0.25 *μ*mol/l) in the absence or presence of GLP-1 (100 nmol/l). Caspase 3/7 enzyme activity was evaluated by using Caspase-Glo 3/7 Assay (Promega, Madison, WI, USA) according to manufacturer’s protocol.

In addition, apoptosis was quantitatively detected by the surface exposure of phosphatidyl serine in apoptotic cells by using an Annexin V-PE and 7-Amino-actinomycin D (7-AAD) double coloration (BD Biosciences, Labware, Bedford, MA). Briefly, (5 x 10^5^ cells/well) INS-1E transfected cells were seeded in six-well and grown for 72 hours. The cells were switched to serum free medium for 12 hours and then incubated (2 hours at 37°C) with staurosporine (0.25 μmol/l) in the absence or presence of GLP-1 (100 nmol/l). Cells were washed and suspended at a concentration of 1 x 10^6^ cells/ml in Annexin V Binding Buffer and then incubated with annexin V and the vital dye 7-AAD for 15 min at room temperature. Apoptosis was assessed using a FACS calibur flow cytometry (Becton Dickinson, San Jose, CA, USA). Annexin V staining was done in conjunction with the vital dye 7-AAD to differentiate early apoptosis (annexin V^+^, 7-AAD^-^) from late apoptosis (annexin V^+^, 7-AAD^+^).

### Western blot analysis

For Western Blot (WB) analysis, cells were seeded in 60-mm dishes at a density of 2 x 10^6^ cells, grown for 72 hours and then serum-starved (12 hours, at 37°C). Cells were then incubated (2 hours at 37°C) in the absence or presence of GLP-1 (100 nmol/l), lysed in ice-cold lysis buffer and protein concentration determined by BCA. Proteins (30μg) were then separated on SDS-polyacrylamide gel and membrane blocked with 5% non-fat dried milk (1 h room temperature) and incubated with primary (1:1000, O/N, 4°C) and then with secondary antibodies (1:2000, 1h, room temperature). Peroxidase activity was detected by films using an enhanced chemiluminescence (ECL) detection reagent (GE Healthcare Life Science).

### Statistical analysis

Results are expressed as means ± S.D. for *n* independent experiments. Statistical differences between groups were evaluated by Student’s t test for unpaired comparison or by ANOVA, as appropriate. A p value less than 0.05 was considered to be significant.

## Results

### GLP-1 effect on insulin secretion

In INS-1E-neo cells, insulin secretion was clearly increased by high glucose concentration (i.e. 16.6 mmol/l as compared to 2.8 mmol/l glucose, considered as the baseline condition, Fig 1A). Under both low and high glucose concentrations, insulin secretion was more than doubled by 100 nmol/l GLP-1 co-incubation (Fig 1A). In INS1-E-ENPP1-cells (see methods and Fig 1A, S1 Fig for the effect of transfection experiments), in which insulin receptor signaling is affected [19], glucose-induced insulin secretion and GLP-1 stimulatory effect were totally blunted, with virtually no differences on insulin secretion across the four different experimental conditions (Fig 1A).

Although some difference was observed in the baseline (2.8 mM glucose) condition, very similar results were obtained at 16.6 mM glucose in a second cell line, namely mouse βTC-1 (Fig 1B), thus showing that our finding was not restricted only to rat beta cells.

### GLP-1 effect on beta cell proliferation

**Fig 2:**
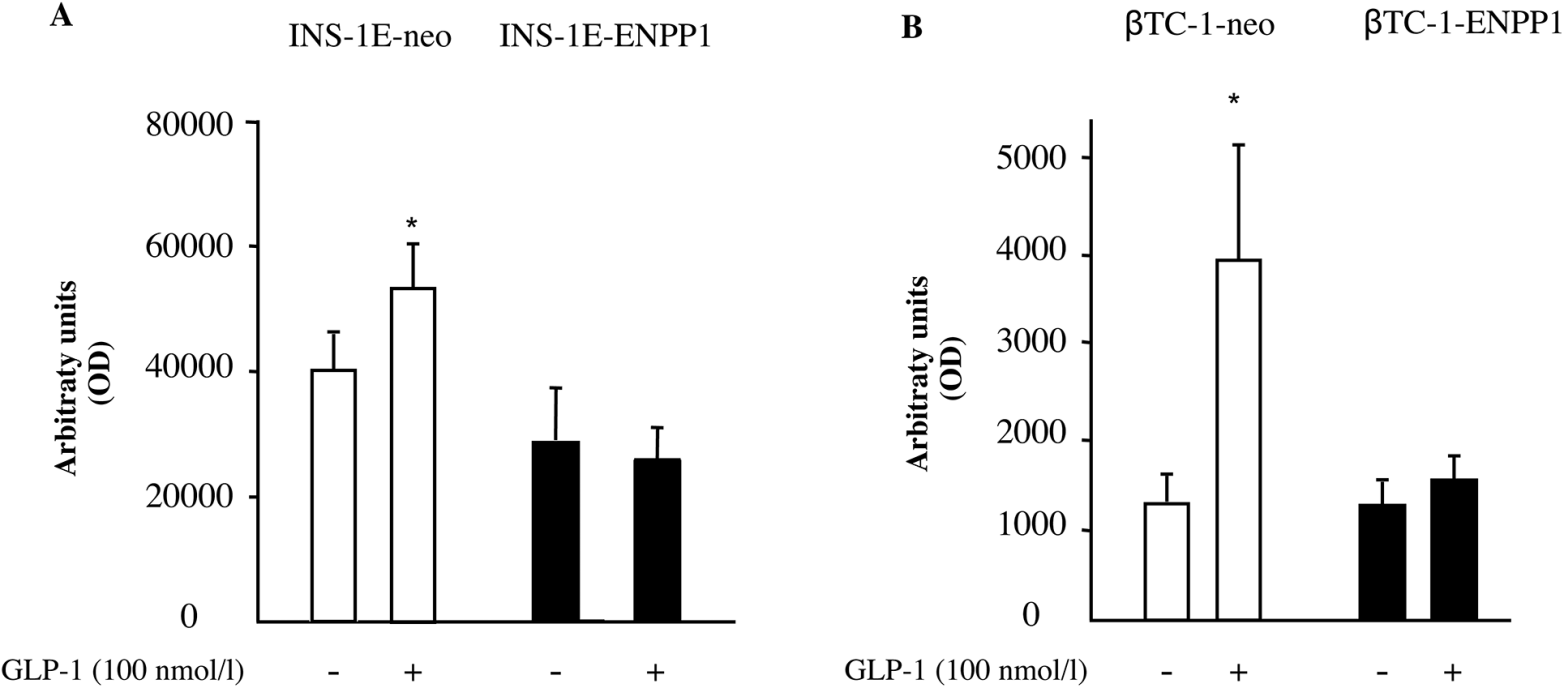
GLP-1 fails to increase cell proliferation in ENPP-1 transfected cells. Proliferation was assessed by BrdU incorporation in INS-1E (panel A) and βTC-1 (panel B) cells, either transfected with neo or ENPP-1 gene variant. Cells were starved for 12 hours and then incubated for 48 hours in the absence or presence of 100 nmol/l GLP-1. Values are means ± S.D. of four independent experiments. * p<0.05 for INS-1E and for βTC-1 compared with untreated INS-1E-neo or βTC-1-neo cells.

In INS-1E-neo cells, GLP-1 (100 nmol/l) significantly increased DNA synthesis but this effect was totally abolished in INS-1E-ENPP1 cells (Fig 2A).

Similarly, 100 nmol/l GLP-1 significantly increased DNA synthesis in βTC-1-neo cells, while no effect at all was observed in βTC-1-ENPP1 cells (Fig 2B).

### GLP-1 effect on apoptosis

**Fig 3:**
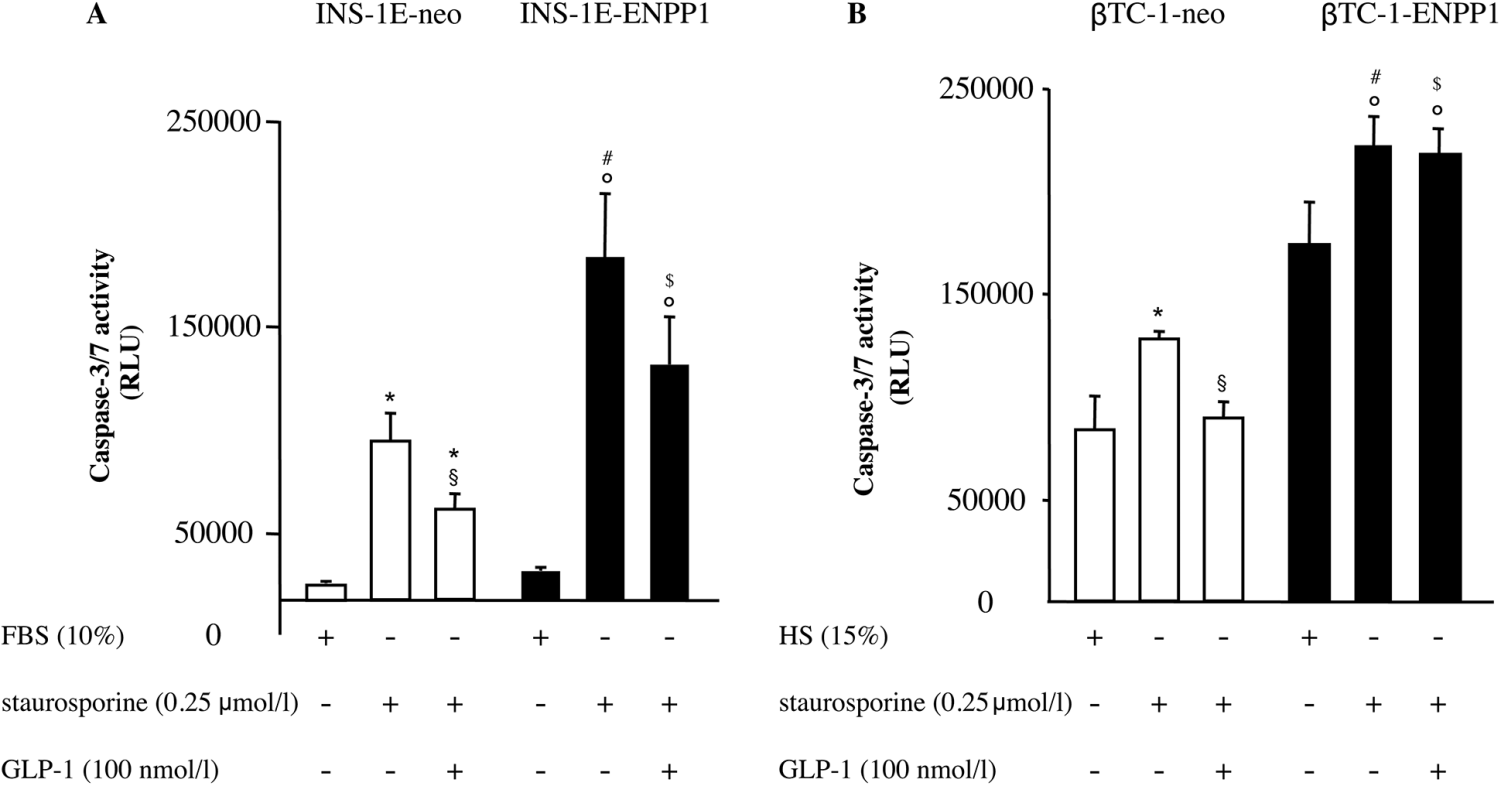
GLP-1 fails to rescue apoptosis in ENPP1 transfected cells. Apoptosis was assessed by caspase 3/7 activity, in INS-1E (panel A) and βTC-1 (panel B) cells, either transfected with neo or ENPP-1 gene variant. Cells were treated with staurosporine (0.25 μmol/l, 2 hours) in the absence or presence of 100 nmol/l GLP-1. Treatment with GLP-1 significantly decreased apoptosis induced by staurosporine in INS-1E-neo and in βTC-1-neo cells but not in INS-1E-ENPP1 and in βTC-1-ENPP1 cells. Values are expressed as means ± SD in INS-1E (n = 7) in βTC-1 (n = 8) independent experiments, each comprising three wells. (* p<0.05 compared with untreated INS-1E-neo or βTC-1-neo cells; § p<0.005 compared with INS-1E-neo or βTC-1-neo cells treated with staurosporine; ° p<0.05 compared with untreated INS-1E-ENPP1 or βTC-1-ENPP1; # p< 0.05 compared with INS-1E-neo or βTC-1-neo cells treated with staurosporine; $ p< 0.05 compared with INS-1E-neo or βTC-1-neo cells exposed to GLP1).

In INS-1E-neo cells, staurosporine treatment (0.25 μmol/l, 2 hours) greatly increased 3/7 caspase activity (Fig 3A). This deleterious effect was significantly (p<0.05) reduced by 100 nmol/l GLP-1 (Fig 3A). Quite notably, in INS-1E-ENPP1 cells, staurosporine deleterious effect on apoptosis was markedly increased and only mildly and not significantly reduced by 100 nmol/l GLP-1 (Fig 3A). Overall, caspase 3/7 activity was significantly increased in ENPP1-cells in respect to their counterpart neo-cells (p<0.05 for all three experimental conditions). Similar results were obtained by using FACS analysis, with higher baseline apoptosis (p<0.05) and no significant GLP-1 anti-apoptotic effect observed in ENPP1-, as compared to neo-cells (data not shown).

Even more striking were the results obtained in βTC-1 cells with staurosporine-induced apoptosis being higher and GLP1-1 protective effect being heavily blunted in ENPP1-cells as compared to neo-cells (Fig 3B). Of note, as a whole, βTC-1 cells showed a much higher baseline level of apoptosis than INS-1E cells.

### GLP-1 effects on insulin secretion, proliferation and apoptosis in insulin receptor knockdown cells

In order to get deeper insights about the role of insulin receptor signaling on GLP-1 effects in insulin secreting beta cells, INS-1E cells were transfected with insulin receptor siRNA, which caused a significant reduction of insulin receptor expression levels (-83±5% p<0.001; see Fig 1C, S1 Fig for the effect of transfection experiments).

**Fig 4:**
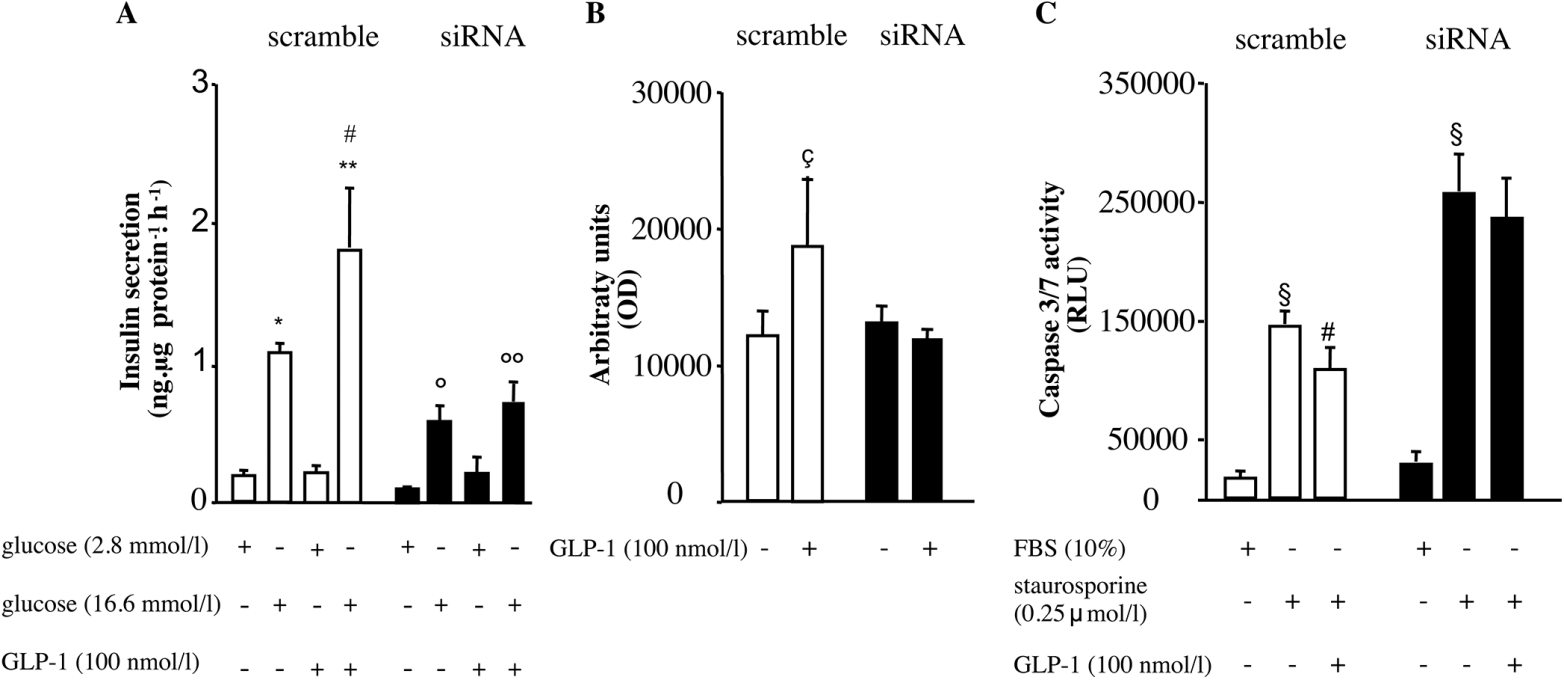
GLP-1 effects on insulin secretion, proliferation and apoptosis are impaired in insulin receptor knockdown cells. Panel A. Insulin secretion in INS-1E cells, transfected with insulin receptor siRNA. Cells were incubated with 2.8 or 16.6 mmol/l glucose in the absence or presence of GLP-1 (100 nmol/l). Values are expressed as means ± SD of three independent experiments (*p<0.05 vs. scramble cells exposed to 2.8 mmol/l glucose; ** p<0.05 vs. scramble cells exposed to 2.8 mmol/l glucose plus 100 nmol/l GLP-1; #vs. scramble cells exposed to 16.6 mmol/l glucose; °p<0.05 vs. siRNA cells exposed to 2.8 mmol/l glucose; °° siRNA cells exposed to 2.8 mmol/l glucose plus 100 nmol/l GLP-1). Panel B. Cell proliferation, assessed by BrdU incorporation, in INS-1E cells transfected with insulin receptor siRNA. Cells were switched to serum free medium for 12 hours and then incubated for 48 hours in the absence or presence of GLP-1 (100 nmol/l). Values are expressed as means ± SD of four independent experiments, each comprising three wells (ç p<0.05 vs. untreated scramble cells). Panel C. Apoptosis, assessed by caspase 3/7 activity, in INS-1E cells transfected with insulin receptor siRNA. Cells were treated with staurosporine (0.25μmol/l, 2 hours) in the absence or presence of 100 nmol/l GLP-1. Treatment with GLP-1 significantly decreased apoptosis induced by staurosporine in scramble control cells but not in siRNA transfected cells. Values are expressed as means ± SD of nine independent experiments, each comprising three wells. (§ p<0.05 vs. untreated scramble or siRNA transfected cells; # p<0.05 vs. scramble cells treated with staurosporine in the absence of GLP-1).

In contrast to scramble control cells, insulin receptor siRNA cells showed no stimulatory 100 nmol/lGLP-1 effect on 16.6 mmol/l glucose-induced insulin secretion (Fig 4A).

Furthermore, while 100 nmol/l GLP-1 significantly increased DNA synthesis in scramble cells, no such effect was observed in insulin receptor SiRNA transfected cells (Fig 4B).

Finally, a significant anti-apoptotic effect of 100 nmol/l GLP-1 was observed in scramble but not in insulin receptor siRNA cells (Fig 4C). It is of note that, in insulin receptor siRNA cells, caspase 3/7 activity was significantly higher than in scramble control cells under FBS, staurosporine alone and staurosporine in the presence of GLP-1 100 nmol/l (p<0.05 for all three conditions).

Data obtained in insulin receptor down-regulated cells strongly reinforce those in cells overexpressing ENPP1 and altogether demonstrate that insulin receptor signaling is critical for the protective GLP-1 effect on beta-cell function and survival.

### GLP-1 intracellular signaling

In order to get some insights on the mechanisms underlying the influence of insulin receptor down regulation on GLP-1 effects on insulin secreting beta cells, AKT and ERK phosphorylation was investigated in INS-1E cell, under several conditions.

**Fig 5:**
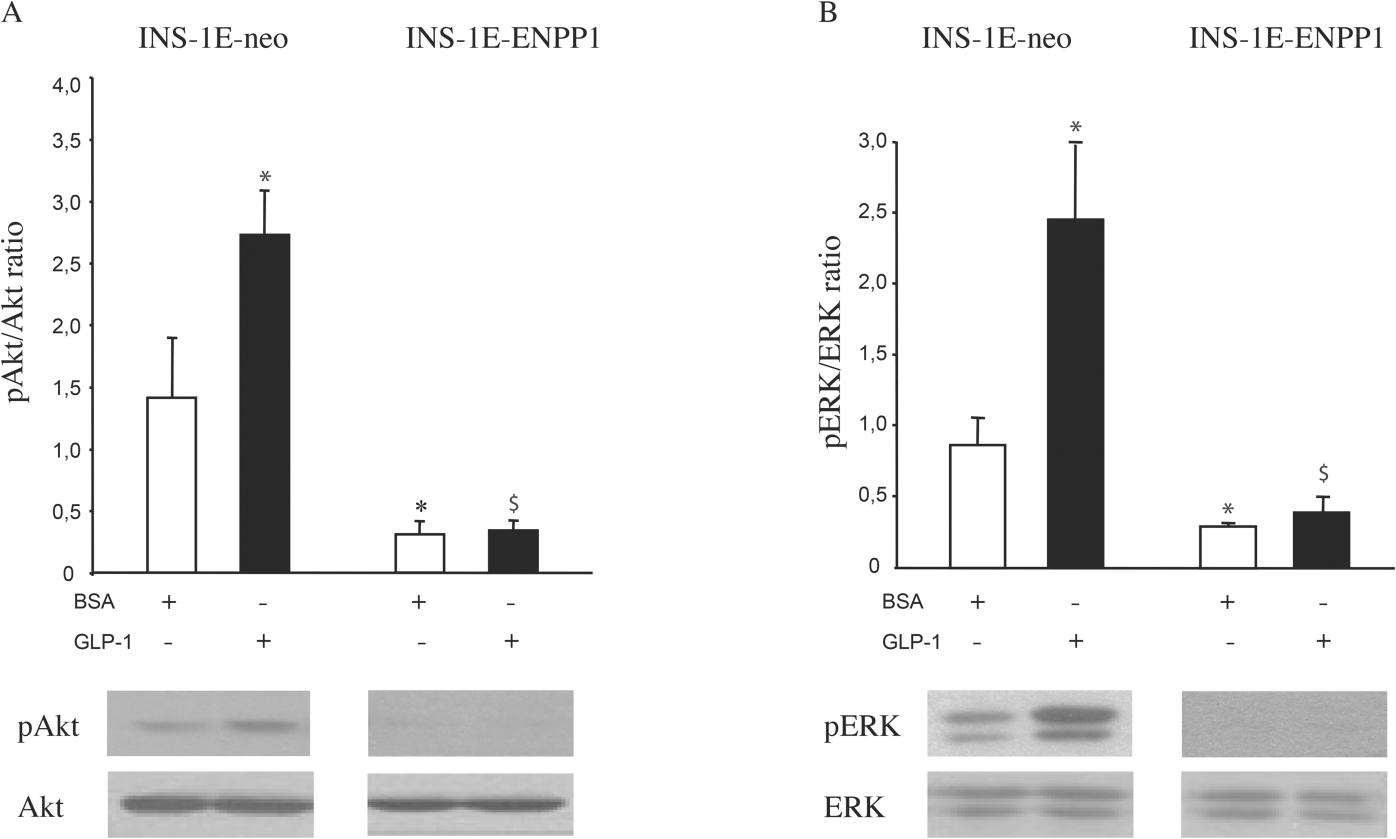
GLP-1 effects on phosphorylation of Akt and ERK 1/2. Phosphorylation of Akt and p-Akt/Akt ratio (panel A) and ERK 1/2 and the p-ERK/ERK ratio (panel B) was evaluated by western blot analysis in INS-1E. Cells were serum-starved for 12 hours and then incubated (2 hours at 37°C) in the presence of BSA or GLP-1 (100 nmol/l). Values are means ± S.D. of four independent experiments. (* p<0.05 compared with BSA treated INS-1E-neo cells; $ vs INS-1E neo GLP-1 100 nM).

Fig 5, panel A, shows that, as compared to unstimulated INS-1E neo-cells, AKT phosphorylation was markedly increased by GLP-1 100 nmol/l stimulation (88% increase, p < 0.05). Conversely, in INS-1E ENPP1-cells, in which baseline AKT phosphorylation was markedly reduced, GLP-1-effect was totally abolished.

Very similar results were obtained when ERK phosphorylation was studied (Fig 5, panel B). Notably, no changes in GLP-1 receptor protein expression were observed, across the same experimental conditions (S1 Fig).

Taken together, these data strongly suggest that the effect of insulin receptor signaling on GLP-1 effects is mainly operating at GLP-1 post-receptor signaling level.

## Discussion

We have previously reported that rat cultured insulin secreting beta cells overexpressing ENPP1, an insulin receptor inhibitor, are characterized by abnormal insulin receptor signaling as well as reduced glucose-induced insulin secretion and cell survival [19]. Such deleterious effects are especially observable when the Q121 gain-of-function variant is operating [19]. In this study we went further on the role of insulin signaling on pancreatic beta cell activities and, by using the same naturally occurring, biological tool (i.e. ENPP1-Q121), report for the first time that an integral insulin receptor signaling is essential for the protective effects of GLP-1 on insulin secretion, cell proliferation and survival to be fully exerted. Identical conclusions can be drawn by experiments in which insulin receptor down-regulation was obtained by small interfering RNA transfection.

The exact mechanisms through which insulin signaling exerts such a permissive effect on GLP-1 action remains elusive. Recent studies have shown that signal transduction, initiated by G-protein coupled receptors and tyrosine-kinase receptors, is organized in mutually related signaling cassettes, leading to a crosstalk between the two signaling pathways [24]. As a matter of fact, our data showing that intact insulin receptor signaling is essential for GLP-1 post-receptor signaling is totally compatible with this scenario. Other studies reported that GLP-1 increases beta cell proliferation not only by activation of GLP-1R receptor but also by up-regulation of IGF-1 receptor expression [22] and transactivation of EGF receptor [23]. Finally, there were evidences of a critical role of IRS2 signaling in promoting exendin-4 stimulated beta cell growth [26]. With this background in mind, it remains possible that the inhibitory activity of reduced insulin signaling on GLP-1 effects we here observed is either directly related to GLP-1 mechanisms of action or, conversely, referable to the more general deleterious effect on several aspects of beta cells functionality we detected also in the absence of GLP-1.

Our present data are somehow in contrast to those of Moon et al. [27] showing that insulin signaling pathway exerts a fine negative tuning on beta cell responsiveness to GLP-1 action. The reasons for such discrepancy are not easy to understand. We can here only speculate that differences in the experimental design for obtaining abnormalities of the insulin signaling pathways, with chemicals whose specificity is far to be demonstrated being utilized in Moon’s study [27], have played a role.

Our study has both strengths and limitations. Among the formers is that though two totally different established tools [27] were used to affect insulin receptor, very similar, virtually superimposable, results have been obtained. A further strength is that wide range of GLP-1 protective effects have been investigated with the deleterious effect of abnormal insulin signaling being observed across all of them. Finally, it is also of note that the most crucial experiments were carried out also in a second cell line, of mouse rather than rat origin, thus making possible to exclude that our findings are either cell- or even species- specific and conversely suggesting they are generalizable to many experimental conditions.

Among limitations we acknowledge that no mechanistic insights, underlying the deleterious effect of abnormal insulin signaling on GLP-1 protective effect on beta cell, have been addressed. In fact, further studies are ongoing in our laboratory aimed at understanding the intimate molecular mechanisms through which insulin signaling exerts its permissive effect on GLP-1 action. Finally, it is unknown if the permissive effect of insulin receptor function on GLP-1 action we observed in beta cells is similarly operating in other cell types, so to hypothesize that defective insulin receptor activity affects other important GLP-1 effects, including that on CNS and hearth [28, 29]. In conclusion our data indicate that the overexpression of ENPP1 (an inhibitor of insulin receptor signaling) as well as the down-regulation of insulin receptor itself, blunt GLP-1 action on beta cell insulin secretion, proliferation and survival, thus providing evidence that integral insulin receptor function is a prerequisite for such GLP-1 positive effects to be fully expressed. Further studies are needed to understand whether this phenomenon plays a role of clinical relevance in the not negligible proportion of diabetic patients experiencing GLP-1 related treatments failure (4–8). More specifically, it will be of note to try understand whether individuals carrying the ENPP1-Q121 variant (i.e. approximately 30% of the whole population) are more prone to be poor responders to such treatments, thus allowing to envisage a precision medicine approach for the treatment of type 2 diabetes mellitus.

## Supporting information

S1 Fig Expression of ENPP1, insulin receptor and GLP-1 receptor. Panel A. ENPP1 expression in INS-1E rat beta cells, evaluated by SDS PAGE followed by immunoblotting with an anti ENPP1 polyclonal antibody.
Panel B. ENPP1 expression in βTC-1 mouse beta cells, evaluated by SDS PAGE followed by immunoblotting with an anti ENPP1 polyclonal antibody. A representative experiment of three independent ones is shown.
Panel C. Insulin receptor expression in INS-1E rat beta cells, transfected with no targeting siRNA (scramble) or specific insulin receptor siRNA (siRNA) evaluated by SDS PAGE followed by immunoblotting with an anti insulin receptor polyclonal antibody. A representative experiment of three independent ones is shown.
Panel D. GLP-1 receptor expression in INS-1E cells transfected with Neo or ENPP1 evaluated by SDS PAGE followed by immunoblotting with an anti GLP-1 receptor monoclonal antibody. A representative experiment of three independent ones is shown.
(TIF)

## References

[1] Drucker DJ. The biology of incretin hormones. Cell Metab 2006; 3(3): 153–65. doi: 10.1016/j.cmet.2006.01.004 16517403 10.1016/j.cmet.2006.01.004

[2] Li Y, Hansotia T, Yusta B, Ris F, Halban PA, Drucker DJ. Glucagon-like peptide-1 receptor signaling modulates beta cell apoptosis. J Biol Chem 2003; 278(1): 471–8. doi: 10.1074/jbc.M209423200 12409292 10.1074/jbc.M209423200

[3] Brubaker PL and Drucker DJ. Glucagon-like peptides regulate cell proliferation and apoptosis in the pancreas, gut, and central nervous system. Endocrinology 2004; 145(6):2653–9. doi: 10.1210/en.2004-0015 15044356 10.1210/en.2004-0015

[4] Khan M, Ouyang J, Perkins K, Nair S, Joseph F. Determining Predictors of Early Response to Exenatide in Patients with Type 2 Diabetes Mellitus. J Diabetes Res 2015; 2015:162718 doi: 10.1155/2015/162718 25688374 10.1155/2015/162718 PMC4320857

[5] Song SO, Kim KJ, Lee BW, Kang ES, Cha BS, Lee HC. Tolerability, effectiveness and predictive parameters for the therapeutic usefulness of exenatide in obese, Korean patients with type 2 diabetes. J Diabetes Investig 2014; 5: 554–562. doi: 10.1111/jdi.12184 25411624 10.1111/jdi.12184 PMC4188114

[6] Kozawa J, Inoue K, Iwamoto R, Kurashiki Y, Okauchi Y, Kashine S, et al Liraglutide is effective in type 2 diabetic patients with sustained endogenous insulin secreting capacity. J Diabetes Investig 2012; 3:294–7. doi: 10.1111/j.2040-1124.2011.00168.x 24843579 10.1111/j.2040-1124.2011.00168.x PMC4014952

[7] Anichini R, Cosimi S, Di Carlo A, Orsini P, De Bellis A, Seghieri G, et al Gender difference in response predictors after 1-year exenatide therapy twice daily in type 2 diabetic patients: a real world experience. Diabetes Metab Syndr Obes: 2013; 6: 123–129 doi: 10.2147/DMSO.S42729 23630427 10.2147/DMSO.S42729 PMC3626369

[8] Shin J, Chang JS, Kim HS, Ko SH, Cha BY, Son HY, et al Effects of a 6-month exenatide therapy on hba1c and weight in Korean patients with type 2 diabetes: a retrospective cohort study. Diabetes and Metab J, 2012; 36: 364–370 23130321 10.4093/dmj.2012.36.5.364 PMC3486983

[9] Kulkarni RN, Brüning JC, Winnay JN, Postic C, Magnuson MA, Kahn CR. Tissue-specific knockout of the insulin receptor in pancreatic beta cells creates an insulin secretory defect similar to that in type 2 diabetes. Cell 1999; 96: 329–39. 10025399 10.1016/s0092-8674(00)80546-2

[10] Kulkarni RN, Winnay JN, Daniels M, Brüning JC, Flier SN, Hanahan D, et al Altered function of insulin receptor substrate-1-deficient mouse islets and cultured beta-cell lines. J Clin Invest 1999; 104: 69–75. 10.1172/JCI8339 PMC409887 10606633

[11] Garofalo RS, Orena SJ, Rafidi K, Torchia AJ, Stock JL, Hildebrandt AL, et al Severe diabetes, age-dependent loss of adipose tissue, and mild growth deficiency in mice lacking Akt2/PKB beta. J Clin Invest 2003; 112: 197–208. doi: 10.1172/JCI16885 12843127 10.1172/JCI16885 PMC164287

[12] Pizzuti A, Frittitta L, Argiolas A, Baratta R, Goldfine ID, Bozzali M, et al A polymorphism (K121Q) of the human glycoprotein PC-1 gene coding region is strongly associated with insulin resistance. Diabetes 1999; 48: 1881–4. 10480624 10.2337/diabetes.48.9.1881

[13] Costanzo BV, Trischitta V, Di Paola R, Spampinato D, Pizzuti A, Vigneri R, et al The Q allele variant (GLN121) of membrane glycoprotein PC-1 interacts with the insulin receptor and inhibits insulin signaling more effectively than the common K allele variant (LYS121). Diabetes 2001; 50(4): 831–6. 11289049 10.2337/diabetes.50.4.831

[14] Federici M, Pandolfi A, De Filippis EA, Pellegrini G, Menghini R, Lauro D, et al G972R IRS-1 variant impairs insulin regulation of endothelial nitric oxide synthase in cultured human endothelial cells. Circulation 2004; 109: 399–405. doi: 10.1161/01.CIR.0000109498.77895.6F 14707024 10.1161/01.CIR.0000109498.77895.6F

[15] Prudente S, Hribal ML, Flex E, Turchi F, Morini E, De Cosmo S, et al The functional Q84R polymorphism of mammalian Tribbles homolog TRB3 is associated with insulin resistance and related cardiovascular risk in Caucasians from Italy. Diabetes 2005; 54: 2807–11. 16123373 10.2337/diabetes.54.9.2807

[16] Prudente S, Morini E, Marselli L, Baratta R, Copetti M, Mendonca C, et al Joint effect of insulin signaling genes on insulin secretion and glucose homeostasis. J Clin Endocrinol Metab 2013; 98: E1143–7. doi: 10.1210/jc.2012-4282 23633196 10.1210/jc.2012-4282 PMC6618023

[17] Marchetti P, Lupi R, Federici M, Marselli L, Masini M, Boggi U, et. al Insulin secretory function is impaired in isolated human islets carrying the Gly(972)—>Arg IRS-1 polymorphism. Diabetes 2002; 51: 1419–24. 11978638 10.2337/diabetes.51.5.1419

[18] Liew CW, Bochenski J, Kawamori D, Hu J, Leech CA, Wanic K, et al The pseudokinase tribbles homolog 3 interacts with ATF4 to negatively regulate insulin exocytosis in human and mouse beta cells. J Clin Invest 2010; 120: 2876–88. doi: 10.1172/JCI36849 20592469 10.1172/JCI36849 PMC2912176

[19] Di Paola R, Caporarello N, Marucci A, Dimatteo C, Iadicicco C, Del Guerra S, et al ENPP1 affects insulin action and secretion: evidences from in vitro studies. PLoS One 2011; 6: e19462 doi: 10.1371/journal.pone.0019462 21573217 10.1371/journal.pone.0019462 PMC3088669

[20] Kahn CR. Knockout mice challenge our concepts of glucose homeostasis and the pathogenesis of diabetes. Exp Diabesity Res 2003; 4(3): 169–82. doi: 10.1155/EDR.2003.169 15061645 10.1155/EDR.2003.169 PMC2478605

[21] Prudente S, Morini E, Trischitta V. Insulin signaling regulating genes: effect on T2DM and cardiovascular risk. Nat Rev Endocrinol 2009; 5(12): 682–93. doi: 10.1038/nrendo.2009.215 19924153 10.1038/nrendo.2009.215

[22] Cornu M, Modi H, Kawamori D, Kulkarni RN, Joffraud M, Thorens B. Glucagon-like peptide-1 increases beta-cell glucose competence and proliferation by translational induction of insulin-like growth factor-1 receptor expression. J Biol Chem 2010; 285(14): 10538–45. doi: 10.1074/jbc.M109.091116 20145256 10.1074/jbc.M109.091116 PMC2856261

[23] Buteau J, Foisy S, Joly E, Prentki M. Glucagon-like peptide 1 induces pancreatic beta-cell proliferation via transactivation of the epidermal growth factor receptor. Diabetes 2003; 52(1): 124–32. 12502502 10.2337/diabetes.52.1.124

[24] Natarajan K, Berk BC. Crosstalk coregulation mechanisms of G protein-coupled receptors and receptor tyrosine kinases. Methods Mol Biol 2006; 332: 51–77. doi: 10.1385/1-59745-048-0:51 16878685 10.1385/1-59745-048-0:51

[25] Patane G, Caporarello N, Marchetti P, Parrino C, Sudano D, Marselli L, et al Adiponectin increases glucose-induced insulin secretion through the activation of lipid oxidation. Acta Diabetol 2013; 50:851–7. doi: 10.1007/s00592-013-0458-x 23440352 10.1007/s00592-013-0458-x

[26] Park S, Dong X, Fisher TL, Dunn S, Omer AK, Weir G, et al Exendin-4 uses Irs2 signaling to mediate pancreatic beta cell growth and function. J Biol Chem 2006; 281: 1159–68. doi: 10.1074/jbc.M508307200 16272563 10.1074/jbc.M508307200

[27] Moon MJ, Kim HY, Park S, Kim DK, Cho EB, Hwang JI, et al Insulin contributes to fine-tuning of the pancreatic beta-cell response to glucagon-like peptide-1. Mol Cells 2011; 32: 389–95. doi: 10.1007/s10059-011-0157-9 21904878 10.1007/s10059-011-0157-9 PMC3887647

[28] Nauck M. Incretin therapies: highlighting common features and differences in the modes of action of glucagon-like peptide-1 receptor agonists and dipeptidyl peptidase-4 inhibitors. Diabetes Obes Metab 2016; 3;18(3):203–16. doi: 10.1111/dom.12591 26489970 10.1111/dom.12591 PMC4785614

[29] Drucker DJ. Deciphering metabolic messages from the gut drives therapeutic innovation: the 2014 Banting Lecture. Diabetes 2015; 64:317–326 doi: 10.2337/db14-1514 25614665 10.2337/db14-1514

