# Supplementary figures and images for: Insulin receptor signaling and glucagon-like peptide 1 effects on pancreatic beta cells
Source: PLoS One. 2017 Aug 2;12(8):e0181190. doi: 10.1371/journal.pone.0181190 (PMC5540605; doi:10.1371/journal.pone.0181190)

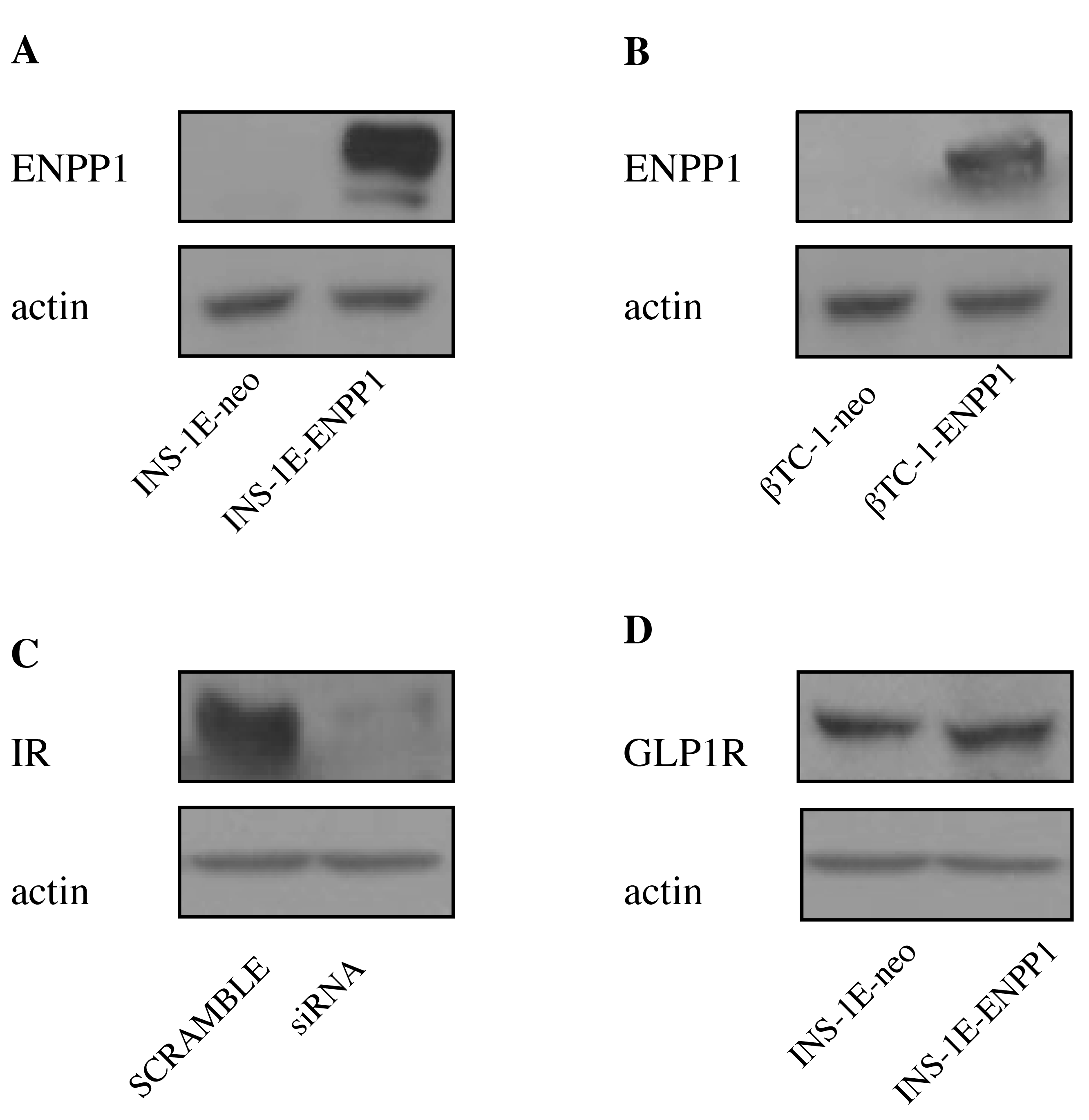

Supplement: S1 Fig — Panel A. ENPP1 expression in INS-1E rat beta cells, evaluated by SDS PAGE followed by immunoblotting with an anti ENPP1 polyclonal antibody. Panel B. ENPP1 expression in βTC-1 mouse beta cells, evaluated by SDS PAGE followed by immunoblotting with an anti ENPP1 polyclonal antibody. A representative experiment of three independent ones is shown. Panel C. Insulin receptor expression in INS-1E rat beta cells, transfected with no targeting siRNA (scramble) or specific insulin receptor siRNA (siRNA) evaluated by SDS PAGE followed by immunoblotting with an anti insulin receptor polyclonal antibody. A representative experiment of three independent ones is shown. Panel D. GLP-1 receptor expression in INS-1E cells transfected with Neo or ENPP1 evaluated by SDS PAGE followed by immunoblotting with an anti GLP-1 receptor monoclonal antibody. A representative experiment of three independent ones is shown. (TIF) [file pone.0181190.s001.tif]
